# Genotyping of *candida albicans* isolates obtained from vulvovaginal candidiasis patients in Zanjan, Iran, based on ABC and RPS typing systems

**DOI:** 10.32598/CMM.2023.1364

**Published:** 2022-12

**Authors:** Saeid Amanloo, Masoomeh Zanjani, Sahar Serajian, Farzaneh Ahmadi, Firoozeh Kakavand

**Affiliations:** 1 Department of Parasitology and Mycology, School of Medicine, Zanjan University of Medical Sciences, Zanjan, Iran; 2 Department of Medical Laboratory Sciences, School of Paramedical Sciences, Zanjan University of Medical Sciences, Zanjan, Iran; 3 Department of Biostatistics and Epidemiology, School of Medicine, Zanjan University of Medical Sciences, Zanjan, Iran; 4 Health and Treatment Center of Zanjan, Zanjan University of Medical Sciences, Zanjan, Iran

**Keywords:** ALT repeat, *Candida albicans*, Genotyping, RPS, 25S rDNA

## Abstract

**Background and Purpose:**

Genotyping of pathogenic microorganisms is important for epidemiological studies and the adoption of appropriate strategies to control infectious diseases. In this regard, the present study aimed to genotype *Candida albicans* strains isolated from vulvovaginal candidiasis (VVC) patients using combined ABC type (25SrDNA) and repetitive sequence (RPS) typing systems. using combined typing systems of ABC type (25SrDNA) and repetitive sequence (RPS).

**Materials and Methods:**

In total, 140 patients with VVC were investigated. Vaginal discharges were collected on Sabouraud dextrose agar and identified by CHROMagar. After species identification, a polymerase chain reaction system targeting 25S rDNA as well as ALT repeats in the RPS was designed to determine *C. albicans* genotypes. The dendrogram was constructed by zero-one matrix data based on the combination of ABC and RPS typing systems. Statistical analysis of data was performed in SPSS software (version 23).

**Results:**

In total, 41 (29.3%) *Candida* isolates were obtained from 140 VVC patients. The most common *Candida* species that were identified included *C. glabrata* (56.1%) and *C. albicans* (39%). Genotype A3 with five isolates (31.25%) had the highest frequency, followed by B2/3 with three isolates (18.3%), A3/4, C3/4, and B3/4 with two isolates (12.5%), and C2/3 and C3 with one isolate (6.25%), respectively. No significant association was found between the genotypes and antifungal resistance (*P*<0.05).

**Conclusion:**

The results showed that non-*albicans Candida* species are more prevalent in VVC patients, compared to *C. albicans*. The results also indicated that ABC and RPS typings are useful for rapid genotyping and differentiation of *C. albicans* isolates in regional and small-scale studies.

## Introduction

There are more than 20 *Candida* species that are known as opportunistic human pathogens. Today, *Candida* infections have increased due to the increase in the use of aggressive medical interventions and the number of immunosuppressed patients. *Candida albicans* is the most important pathogen of the *Candida* genus, which could cause mucocutaneous or systemic infections in healthy people or patients with underlying diseases, such as immunosuppression [ 1 ]. Although *C. albicans* is still known as the main pathogen of this genus, there is a pattern change from *albicans* to non-*albicans* infections, especially *C. glabrata* and *C. tropicalis* infections [ 2 ]. 

One of these infections is vulvovaginal candidiasis (VVC) whose severity and frequency could be different among different people. It has been estimated that 75% of women experience vaginitis at least once in their lifetime which negatively affects their social and work lives [ 3 ]. The VVC is the second most common infection after bacterial vaginitis. *Candida albicans* has been reported as the most common cause of VVC worldwide. However, the increasing prevalence of vaginitis caused by non-*albicans Candida* species is a public health concern due to their tendency to develop resistance to azoles [ 4 - 6 ].

It is necessary to use reliable and reproducible molecular typing methods in epidemiological studies of *Candida* infections. It is possible to genetically classify clinical isolates in terms of drug susceptibility patterns, type of infection, and the site of anatomical involvement based on genetic diversity and polymorphism [ 7 ]. The ABC typing based on ribosomal sequences of the 25S rDNA region has been used in several studies in the genotyping of *C. albicans* isolates into A, B, C, D, and E types [ 8 - 10 ]. 

Repetitive sequences (RPSs) were found in all *C. albicans* chromosomes except for chromosome 3 [ 11 ]. Each RPS region has a short tandem repeating unit of 172 bp, which is known as ALT. The number of ALT repeats in RPS is different for each chromosome, causing variation in RPSs electrophoresis fragment size [ 12 ]. The combined analysis of both ABC and RPS could improve the discriminatory power of genotyping [ 13 ]. 

Epidemiological studies of pathogenic microorganisms are important for the adoption of appropriate strategies to control infectious diseases [ 14 , 15 ]. Given the importance of genotyping in epidemiological studies, ABC and RPS typing systems were applied to determine the genotypes and identify the population structure of *C. albicans* strains isolated from vaginal candidiasis. Moreover, the results were compared with the genotyping profile of isolates obtained from oropharyngeal candidiasis samples in a previous study.

## Materials and Methods

### Strains

In this study, 41 *Candida* colonies were collected from 140 VVC patients referring to three health centers in Zanjan, Iran in 2019. In total, 16 isolates were identified as *C. albicans*. The isolates were evaluated in terms of their genotypes using ABC and RPS genotyping systems. 

### DNA extraction and polymerase chain reaction conditions

The DNA extraction was performed using the conventional phenol-chloroform extraction method [ 16 ]. The ABC typing of *C. albicans* isolates based on the 25s rDNA region sequence was performed using the primer pairs of CA-INT-L and CA-INT-R. Moreover, two further primers, ASDcF and pCSCR, were used to determine genotypes based on ALT repeats [ 14 , 17 ]. Details of the primers and the expected band size of polymerase chain reaction (PCR) products have been described previously [ 13 ]. 

The PCR amplification conditions were similar to those reported in previous studies [ 6 ]. Briefly, the volume of the reaction mixture was 25 µL which consisted of 1 µL (4 ng) of DNA template, 1.5 µL of each forward and reverse primer (5 mM), 12.5 µL of master mix (SinaClon Bioscience Co., Karaj, Iran), and 8.5 µL of distilled water. Thermal cycling started at 97 ˚C for 5 min, followed by 30 cycles of denaturation at 94 ˚C for 30 s, annealing at 60 ˚C for 30 s, extension at 72 ˚C for 40 s, and a final extension at 72 ˚C for 5 min. It should be noted that all reactions were performed using a thermal cycler (SimpliAmp; Applied biosystem; Cat No: A24811). The PCR products were electrophoresis on 1.5% agarose gel in TBE buffer.

### Drug susceptibility testing

Antifungal susceptibility tests to fluconazole (FCZ), itraconazole (ICZ), and ketoconazole (KCZ) (Sigma-Aldrich, St. Louis, MO, USA) were performed using the broth micro-dilution method according to M27-A3 and M27-S4 documents [ 18 , 19 ]. The drug concentration was considered to be 0.5-128 μg/mL for fluconazole and 0.03-16 μg/mL for the other two antifungals.

### Phylogenetic analysis

First, the zero-one matrix was prepared by electrophoresis banding patterns. Phylogenetic analysis was performed based on zero-one matrix data via the unweighted pair group method with arithmetic averages (UPGMA) using an online tool ().

The discriminatory power of ABC and RPS typing systems was measured by Simpson’s diversity index using an online tool () [ 20 ]. This index demonstrates the ability of typing methods in differentiating between species.

### Statistical analysis

Statistical analysis of collected data was carried out in SPSS software (version 23). The associations between genotypes and antifungal susceptibility were evaluated using Fisher's exact test. The *P* values < .05 were considered statistically significant.

## Results

In this study, genotyping of 16 clinical isolates of *C. albicans* was performed by ABC and RPS typing. As shown in Table 1, the obtained PCR products using specific pairs of primers (CA-INT-L and CA-INT-R as well as ASDcF- pCSCR) provided distinct band patterns to differentiate genotypes. In this study, ABC typing of *C. albicans* isolates based on the 25s rDNA region sequence presented three genotypes, namely A, B, and C. Genotype A with seven isolates (43.7%) was the most frequent genotype, followed by genotype B (n=5, 31.3%) and genotype C (n=4, 25%). In addition, the banding pattern of RPS typing showed that genotypes 3 and 3/4 had the highest and equal frequencies (n=6, 37.5%), followed by genotype 2/3 (n=4, 25%). The distribution of genotypes based on the combined ABC and RPS typing is summarized in Table 1.

**Table 1:** Frequency distribution of *Candida albicans* clinical isolates in the combination of the ABC and repetitive sequence (ALT Repeats) genotyping

ABC typing	RPS (ALT repeats)		
	3	2/3	3/4
A	5 (31.3%)	2 (12.5%)	2 (12.5%)
B	0 (0.0%)	3 (18.3%)	2 (12.5%)
C	1 (6.3%)	1 (6.3%)	2 (12.5%)

**Figure 1:**
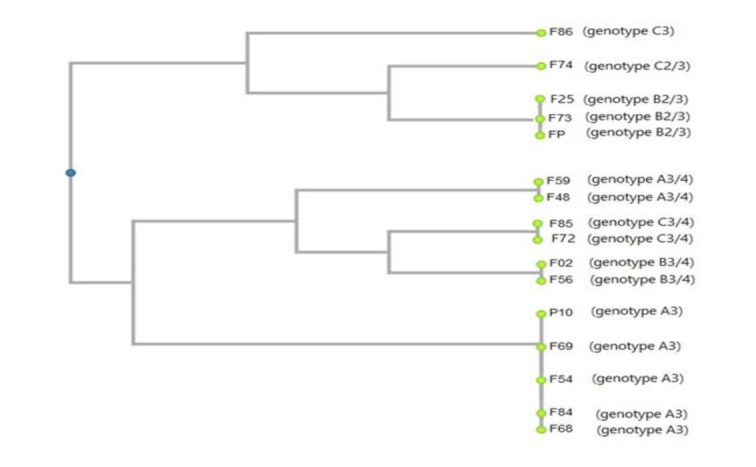
The unweighted pair group method with arithmetic averages dendrogram shows the genetic relationships between 16 isolates of *Candida albicans*. The dendrogram was constructed by zero-one matrix data from combinations of ABC and repetitive sequence typing systems

As shown in Figure 1, the UPGMA dendrogram was constructed based on the combined ABC and RPS (ALT repeats) typing data using an online tool ()

Based on this phylogenetic tree, the studied isolates were divided into seven clades with distinct genotypes. Genotype A3 with five isolates (31.3%) was the most prevalent type, followed by genotype B2/3 (n=3, 18.3%), genotypes A3/4, C3/4, and B3/4 (n=2, 12.5%), and genotypes C3 and C2/3 (n=1, 6.3%).

The discriminatory power of typing systems was calculated according to Simpson’s diversity index using an online tool () [ 20 ]. According to the results, 16 clinical isolates of *C. albicans* were classified into seven distinct genotypes, indicating a discriminatory power index of 0.8667. 

### Antifungal susceptibility testing

*In-vitro* antifungal susceptibility tests to fluconazole, ketoconazole, and itraconazole were performed using the CLSI broth micro-dilution method. These drugs are the most commonly and widely used drugs in cases of vaginal candidiasis. The results of drug sensitivity testing are shown in Table 2.

**Table 2:** No. of clinical isolates of *Candida albicans* according to minimum inhibitory concentration values

Fluconazole (µg/ml)										
0.0625	0.125	0.25	0.5	1	2	4	8	16	32	64
-	1	1	2	1	-	4	2	3	1	1
**Ketoconazole (µg/ml)**										
0.03125	0.0625	0.125	0.25	0.5	1	2	4	8	16	
1	2	1	2	1	2	1	3	2	1	
**Itraconazole (µg/ml)**										
0.03125	0.0625	0.125	0.25	0.5	1	2	4	8	16	
-	1	-	2	1	1	3	3	3	2	

According to the CLSI standard for *C. albicans* isolates, fluconazole minimum inhibitory concentration (MIC) values of ≤ 2, 4, and ≥ 8 µg/mL are considered sensitive, susceptible dose-dependent (SDD), and resistant, respectively. Regarding itraconazole and ketoconazole, MIC values of ≤ 0.12, 0.25-0.5, and ≥ 1 µg/mL are considered sensitive, SDD, and resistant, respectively [ 21 ]. Antifungal susceptibility results indicated that seven (43.75%) isolates were resistant to fluconazole (MIC: ≥8 μg/mL). Besides, resistance to itraconazole and ketoconazole (MIC: ≥1 μg/mL) was observed in 12 (75%) and 9 (56.25%) isolates, respectively. The results of antifungal susceptibility testing of 16 clinical isolates of *C. albicans* are tabulated in Table 3.

**Table 3:** Frequency distribution of *Candida albicans* clinical isolates based on antifungal susceptibility profiles

	Susceptibility	Antifungal drugs		
		Fluconazole	Itraconazole	Ketoconazole
MIC	Sensitive	5 (31.25%)	1 (6.25%)	4 (25%)
	SDD	4 (25%)	3 (18.75%)	3 (18.75%)
	Resistant	7 (43.75%)	12 (75%)	9 (56.25%)

MIC: minimum inhibitory concentration, SDD: susceptible dose-dependent.

## Discussion

In this study, genotyping of clinical isolates of *C. albicans* were conducted based on ABC and RPS typing method. The frequency distribution of *C. albicans* isolates in terms of ABC typing was as follows: genotype A with seven (43.75%) isolates, genotype B with five (31.25%) isolates, and genotype C with four (25%) isolates. *Candida dubliniensis* electrophoretic band pattern was not observed in any of the samples. In addition, the frequency distribution of *C. albicans* isolates in terms of ALT repeats was as follows: ALT3 and ALT 3/4 with six isolates apiece (37.5%) and ALT 2/3 with four isolates (25%). Therefore, genotype A3 with five isolates (31.3%) had the highest frequency. The distribution of genotypes based on the combined ABC and RPS typing is summarized in Table 1. These results are consistent with those of previous studies that have declared genotype A is the dominant genotype [ 6 , 8 , 10 , 13 , 14 , 22 - 24 ]. 

Hattori et al., (2006) reported that the highest frequency of *C. albicans* isolates was related to genotype A3, followed by genotypes A3/4 and B3 [ 8 ]. In a study, a significant association was found between ABC genotypes and blood invasion, and genotype A had a higher prevalence in blood samples [ 22 ]. Iwata et al. (2006) in their study performed in Japan found that among 179 clinical isolates, genotype A with 92 isolates had the highest frequency, followed by genotypes B and C with 38 and 6 isolates, respectively. They also found that based on ALT repeat typing, type three had the highest frequency [ 13 ]. However, the frequency rates of genotypes A, B, and C vary in different studies which could be due to the diversity of isolates in different studies. Several studies have suggested that genotypic differences in *C. albicans* isolates may be related to the site of infection [ 25 - 30 ]. 

In drug susceptibility testing of *Candida* isolates, it was found that drug resistance had no significant correlation with ABC and RPS genotypes, which is in accordance with the results of our previous report [ 6 ]. Studies have shown that there is a genotypic association between ABC genotypes and resistance to flucytosine; accordingly, genotype A is less sensitive and genotype B is more sensitive to flucytosine [ 14 , 29 , 30 ]. 

The isolates obtained from vaginal discharge samples in this study were compared with those obtained from oral lesions in our previous study in terms of the frequency of genotypes [ 6 ]. The results showed that in both studies, genotype A3 had the highest prevalence. The frequency of genotypes B3 and C3 in vaginal discharge samples was zero (0.0%) and one case (6.3%), respectively. Nevertheless, in oral lesion samples, the frequency of B3 and C3 genotypes was six isolates (19.35%) [ 6 ]. Furthermore, 16 isolates from vaginal discharge samples with seven distinct genotypes had a DP of 0.8667, while 31 isolates from oral lesions with seven separate genotypes had a DP of 0.7634. A comparison of the results of these two studies shows that the genotypic diversity is higher in isolates obtained from vaginal discharge samples, compared to those obtained from oral lesions.

Studies have shown that there is no significant correlation between genotypic diversity and the anatomical origins of isolates [ 8 , 31 , 32 ]. Moreover, it has been found that there is no clear genotypic relationship between blood and non-blood isolates of *C. albicans* [ 33 ]. 

Results of a study performed by Hattori et al. (2006) showed that the strains isolated from infectious and non-infectious areas (e.g., mouth and feces) had a similar genotype in each patient. This finding strengthens the possibility of endogenous *C. albicans* infections [ 8 ]. According to the aforementioned report, *C. albicans* has a unique genotype in each person, and this genotype is the same even in different anatomical areas. In that study, no genotypic differences were observed between *C. albicans* strains isolated from the same individual over 6 months [ 8 ]. 

Xiao-dong et al. (2008) used EcoRI and ClaI enzymes to treat the 780 bp PCR product of the RPS region of *C. albicans* strains isolated from skin and vaginal discharge samples using the restriction fragment length polymorphism method [ 34 ]. In their study, 10 distinct genotypes were identified, and no significant genotypic differences were observed between the two groups. However, *C. albicans* strains isolated from different skin regions of each patient had the same genotypic characteristics [ 34 ]. In contrast, Xu et al. (1999) showed that multiple genotypes of the same species could colonize different areas of the host body, indicating the dynamics of the colonization process in the host [ 25 ].

The codon adaptation index gene analysis and single-strand conformational polymorphism genotyping of *C. albicans* isolates have shown that most VVC-associated isolates have specific genotypes [ 35 ]. Moroever, the genotypic distribution of *C. albicans* strains isolated from VVC, balanitis, and non-genital infections was investigated in a study by Li in 2008. The findings revealed that some vaginopathic *C. albicans* isolates exhibited high virulence and vaginal tropism and that the possibility of sexual transmission of vaginal infections was very high. These results clarify the importance of species differences in the etiology of VVC, the relationship between specific genotypes and genital infections, as well as the importance of genotyping in the clinical diagnosis and treatment of VVC. 

Nevertheless, there is no clear correlation between different typing methods, and no reliable genotyping technique has been provided to classify or differentiate pathogenic and drug-resistant strains yet. Nevertheless, genotyping using sequencing techniques, such as multilocus sequence typing, is considered an ideal but costly method with high discriminatory power and clear results [ 36 - 39 ].

## Conclusion

The RPS/ALT typing method is a simple, fast, and affordable method that could be used in epidemiological studies and the management of *Candida* infections. Besides, by using this technique, *C. albicans* could be distinguished from *C. dubliniensis* and *C. stellatoidea* based on the number of ALT repetitions in RPS [ 8 , 13 , 23 ].

## Acknowledgments

The authors would like to appreciate the Research Affairs of Zanjan University of Medical Sciences, Zanjan, Iran for their financial support. They are also very grateful to all the patients, who participated in this study.

## Authors’ contribution

Conceptualization: S. A.; funding acquisition: S. A.; Sample collection: F. K.; laboratory analysis: S. A., M. Z., S. S.; interpretation of the results: S. A., F. A.; writing of the original draft: S. A., M. Z., S. S.; writing review and editing: S. A. 

## Conflicts of interest

There was no conflict of interest between the authors of this study.

## Financial disclosure

This study was financially supported by Zanjan University of Medical Sciences. 

## Ethical Considerations

This study was approved by ethics committee of Zanjan University of Medical Sciences with registration code: ZUMS.REC.1396.192. The patients were informed about the purpose of the study and informed consent was obtained from each participant.

## References

[1] Ciurea CN, Kosovski IB, Mare AD, Toma F, Pintea-Simon IA, Man A ( 2020). Candida and candidiasis-opportunism versus pathogenicity: a review of the virulence traits. Microorganisms.

[2] Friedman DZ, Schwartz IS ( 2019). Emerging fungal infections: new patients, new patterns, and new pathogens. J Fungi.

[3] Tapia CV, Hermosilla G, Fortes P, Alburquenque C, Bucarey S, Salinas H, et al ( 2017). Genotyping and persistence of Candida albicans from pregnant women with vulvovaginal candidiasis. Mycopathologia.

[4] Chong PP, Hadi SR, Lee YL, Phan CL, Tan BC, Ng KP, et al ( 2007). Genotyping and drug resistance profile of Candida spp. in recurrent and one-off vaginitis, and high association of non-albicans species with non-pregnant status. Infect Genet Evol.

[5] Ying C, Zhang H, Tang Z, Chen H, Gao J, Yue C ( 2016). Antifungal susceptibility and molecular typing of 115 Candida albicans isolates obtained from vulvovaginal candidiasis patients in 3 Shanghai maternity hospitals. Med Mycol.

[6] Amanloo S, Katiraee F, Didehdar M, Mohammadi J, Alibabaei Z ( 2020). Genotyping of Candida albicans strains obtained from oropharyngeal candidiasis patients based on abc and rps typing systems. Jundishapur J Microbiol.

[7] Odds FC, Jacobsen MD ( 2008). Multilocus sequence typing of pathogenic Candida species. Eukaryotic cell.

[8] Hattori H, Iwata T, Nakagawa Y, Kawamoto F, Tomita Y, Kikuchi A, et al ( 2006). Genotype analysis of Candida albicans isolates obtained from different body locations of patients with superficial candidiasis using PCRs targeting 25S rDNA and ALT repeat sequences of the RPS. J Dermatol Sci.

[9] Lian C, Zhao J, Zhang Z, Liu W ( 2004). Genotype of Candida species associated with different conditions of vulvovaginal candidosis. Mycoses.

[10] Millar BC, Moore JE, Xu J, Walker MJ, Hedderwick S, McMullan R ( 2002). Genotypic subgrouping of clinical isolates of Candida albicans and Candida dubliniensis by 25S intron analysis. Lett Appl Microbiol.

[11] Hattori H, Tanaka R, Chibana H, Kawamoto F, Adachi H, Shimizu K, et al ( 2009). Improvement of the repetitive sequence-based identification and genotyping of Candida albicans using ALT-specific primers. Jpn J Infect Dis.

[12] Mijiti J, Pu XM, Erfan A, Yaguchi T, Chibana H, Tanaka R ( 2010). Genotyping of fluconazole-resistant Candida albicans isolated from Uighurian people in Xinjing (China) using ALTS/RFLP and micro-TGGE method. Nippon Ishinkin Gakkai Zasshi.

[13] Iwata T, Hattori H, Chibana H, Mikami Y, Tomita Y, Kikuchi A, et al ( 2006). Genotyping of Candida albicans on the basis of polymorphisms of ALT repeats in the repetitive sequence (RPS). J Dermatol Sci.

[14] McCullough MJ, Clemons KV, Stevens DA ( 1999). Molecular and phenotypic characterization of genotypic Candida albicans subgroups and comparison with Candida dubliniensis and Candida stellatoidea. J Clin Microbiol.

[15] Pfaller MA ( 1995). Epidemiology of fungal infections: the promise of molecular typing. Clin Infect Dis.

[16] Müller FM, Werner KE, Kasai M, Francesconi A, Chanock SJ, Walsh TJ ( 1998). Rapid extraction of genomic DNA from medically important yeasts and filamentous fungi by high-speed cell disruption. J Clin Microbiol.

[17] Chibana H, Iwaguchi S, Homma M, Chindamporn A, Nakagawa Y, Tanaka K ( 1994). Diversity of tandemly repetitive sequences due to short periodic repetitions in the chromosomes of Candida albicans. J Bacteriol.

[18] Wayne PA (2008). Reference method for broth dilution antifungal susceptibility testing of yeasts, approved standard.

[19] Wayne PA (2012). Reference method for broth dilution antifungal susceptibility testing of yeasts, approved standard.

[20] Hunter PR ( 1990). Reproducibility and indices of discriminatory power of microbial typing methods. J Clin Microbiol.

[21] Pfaller MA, Diekema D ( 2012). Progress in antifungal susceptibility testing of Candida spp. by use of Clinical and Laboratory Standards Institute broth microdilution methods, 2010 to 2012. J Clin Microbiol.

[22] Karahan ZC, Güriz H, Ağırbaşlı H, Balaban N, Göçmen JS, Aysev D, et al ( 2004). Genotype distribution of Candida albicans isolates by 25S intron analysis with regard to invasiveness. Mycoses.

[23] Tamai IA, Salehi TZ, Sharifzadeh A, Shokri H, Khosravi AR ( 2014). Repetitive sequences based on genotyping of Candida albicans isolates obtained from Iranian patients with human immunodeficiency virus. Iran J Basic Med Sci.

[24] Tamura M, Watanabe K, Mikami Y, Yazawa K, Nishimura K ( 2001). Molecular characterization of new clinical isolates of Candida albicans and C. dubliniensis in Japan: analysis reveals a new genotype of C. albicans with group I intron. J Clin Microbiol.

[25] Xu J, Boyd CM, Livingston E, Meyer W, Madden JF, Mitchell TG ( 1999). Species and genotypic diversities and similarities of pathogenic yeasts colonizing women. J Clin Microbiol.

[26] Sampaio P, Gusmao L, Correia A, Alves C, Rodrigues AG, Pina-Vaz C, et al ( 2005). New microsatellite multiplex PCR for Candida albicans strain typing reveals microevolutionary changes. J Clin Microbiol.

[27] Khatib R, Ramanathan J, Riederer KM, DePoister Jr D, Baran Jr J ( 2002). Limited genetic diversity of Candida albicans in fecal flora of healthy volunteers and inpatients: a proposed basis for strain homogeneity in clinical isolates. Mycoses.

[28] Lockhart SR, Reed BD, Pierson CL, Soll DR ( 1996). Most frequent scenario for recurrent Candida vaginitis is strain maintenance with" substrain shuffling": demonstration by sequential DNA fingerprinting with probes Ca3, C1, and CARE2. J Clin Microbiol.

[29] Stevens DA, Odds FC, Scherer S ( 1990). Application of DNA typing methods to Candida albicans epidemiology and correlations with phenotype. Rev Infect Dis.

[30] Mercure S, Montplaisir S, Lernay G ( 1993). Correlation between the presence of a self-splicing intron in the 25S rDNA of C. albicans and strains susceptibility to 5-fluorocytosine. Nucleic Acids Res.

[31] Millar BC, Xu J, McMullan R, Walker MJ, Hedderwick S, Moore JE ( 2005). Frequency and distribution of group I intron genotypes of Candida albicans colonising critically ill patients. Br J Biomed Sci.

[32] Soll DR, Galask R, Schmid J, Hanna C, Mac K, Morrow B ( 1991). Genetic dissimilarity of commensal strains of Candida spp. carried in different anatomical locations of the same healthy women. J Clin Microbiol.

[33] Adachi H, Shimizu K, Hattori H, Tanaka R, Chibana H, Takagi Y, et al ( 2009). Genotyping of Candida albicans by fragment analysis of microsatellites combined with 25S rDNA and RPS-based strategies. Nippon Ishinkin Gakkai Zasshi.

[34] She XD, Wang XJ, Fu MH, Shen YN, Liu WD ( 2008). Genotype comparisons of strains of Candida albicans from patients with cutaneous candidiasis and vaginal candidiasis. Chin Med J.

[35] Fan SR, Bai FY, Liao QP, Liu ZH, Li J, Liu XP ( 2008). Genotype distribution of Candida albicans strains associated with different conditions of vulvovaginal candidiasis, as revealed by microsatellite typing. Sex Transm Infect.

[36] Bougnoux ME, Morand S, d'Enfert C ( 2002). Usefulness of multilocus sequence typing for characterization of clinical isolates of Candida albicans. J Clin Microbiol.

[37] Dodgson AR, Pujol C, Denning DW, Soll DR, Fox AJ ( 2003). Multilocus sequence typing of Candida glabrata reveals geographically enriched clades. J Clin Microbiol.

[38] Robles JC, Koreen L, Park S, Perlin DS ( 2004). Multilocus sequence typing is a reliable alternative method to DNA fingerprinting for discriminating among strains of Candida albicans. J Clin Microbiol.

[39] Tavanti A, Gow NA, Senesi S, Maiden MC, Odds FC ( 2003). Optimization and validation of multilocus sequence typing for Candida albicans. J Clin Microbiol.

